# Identification of a Transcriptomic Prognostic Signature by Machine Learning Using a Combination of Small Cohorts of Prostate Cancer

**DOI:** 10.3389/fgene.2020.550894

**Published:** 2020-11-25

**Authors:** Benjamin Vittrant, Mickael Leclercq, Marie-Laure Martin-Magniette, Colin Collins, Alain Bergeron, Yves Fradet, Arnaud Droit

**Affiliations:** ^1^Centre de Recherche du CHU de Québec – Université Laval, Québec, QC, Canada; ^2^Département de Médecine Moléculaire, Université Laval, QC, Canada; ^3^Universities of Paris Saclay, Paris, Evry, CNRS, INRAE, Institute of Plant Sciences Paris Saclay (IPS2), 91192, GIf sur Yvette, France; ^4^UMR MIA-Paris, AgroParisTech, INRA, Université Paris-Saclay, Paris, France; ^5^Vancouver Prostate Cancer Centre, Vancouver, BC, Canada; ^6^Department of Urologic Sciences, The University of British Columbia, Vancouver, BC, Canada; ^7^Département de Chirurgie, Oncology Axis, Université Laval, Québec, QC, Canada

**Keywords:** machine learning, prostate cancer, RNA-seq, biochemical recurrence, random forest, predictive signature

## Abstract

Determining which treatment to provide to men with prostate cancer (PCa) is a major challenge for clinicians. Currently, the clinical risk-stratification for PCa is based on clinico-pathological variables such as Gleason grade, stage and prostate specific antigen (PSA) levels. But transcriptomic data have the potential to enable the development of more precise approaches to predict evolution of the disease. However, high quality RNA sequencing (RNA-seq) datasets along with clinical data with long follow-up allowing discovery of biochemical recurrence (BCR) biomarkers are small and rare. In this study, we propose a machine learning approach that is robust to batch effect and enables the discovery of highly predictive signatures despite using small datasets. Gene expression data were extracted from three RNA-Seq datasets cumulating a total of 171 PCa patients. Data were re-analyzed using a unique pipeline to ensure uniformity. Using a machine learning approach, a total of 14 classifiers were tested with various parameters to identify the best model and gene signature to predict BCR. Using a random forest model, we have identified a signature composed of only three genes (JUN, HES4, PPDPF) predicting BCR with better accuracy [74.2%, balanced error rate (BER) = 27%] than the clinico-pathological variables (69.2%, BER = 32%) currently in use to predict PCa evolution. This score is in the range of the studies that predicted BCR in single-cohort with a higher number of patients. We showed that it is possible to merge and analyze different small and heterogeneous datasets altogether to obtain a better signature than if they were analyzed individually, thus reducing the need for very large cohorts. This study demonstrates the feasibility to regroup different small datasets in one larger to identify a predictive genomic signature that would benefit PCa patients.

## Introduction

Prostate Cancer (PCa) is the most common non-cutaneous cancer in American men. Around 160 000 men were diagnosed with PCa in 2017 (Siegel et al., 2017) and around 27 000 died of it. The burden of this disease on public health is important and expected to grow as a recent study revealed that the incidence of advanced PCa increased in the last few years (Weiner et al., 2016). PCa is a complex and heterogeneous disease (D’Amico et al., 2003; Buyyounouski et al., 2012) since the risk of relapse and death after treatment differs among cancers with the same clinico-pathological features, namely the grade (Gleason score), stage [Tumor, Node, Metastasis (TNM)] (Edge and Compton, 2010; Amin et al., 2018) and the level of prostatic specific antigen (PSA) (Papsidero et al., 1980).

Current treatments for localized PCa mainly include surgical removal or external beam radiation therapy of the prostate. If the initial treatments did not succeed to cure the patient then a recurrence will occur, revealed by an increase in seric PSA level, an event called biochemical recurrence (BCR). After surgery, about 70% of the patients will be cured and about 30% will relapse to a BCR. Since prostate tumor cells depend on androgens to grow, recurrences are treated with androgen deprivation therapy consisting in chemical or surgical castration either alone or in association with administration of anti-androgens. However, the cancer will inevitably recur and will then be called castration-resistant prostate cancer (CRPC). To treat CRPC, docetaxel (Tannock et al., 2004) was introduced in 2004, but more recently, second generation of androgen-deprivation therapies resulted in better survival (Tannock et al., 2004; Nevedomskaya et al., 2018). Ultimately all these tumors will relapse and patients will be offered palliative therapy. Consequently, in order to offer better treatments to these patients, there is a pressing need to identify earlier those tumors that will recur after surgery and evolve to become lethal.

One problem generally inherent to cancer care is to orient people to the adequate treatment corresponding to the stage of the disease and the individual characteristics of the patient (Terada et al., 2017). In PCa, the stage, grade and PSA level are currently the best standards to drive patients in the different treatment options. Currently, after radical prostatectomy the PSA level is actively monitored to assess the BCR, but there is no biomarker that is used clinically to predict a future BCR.

To reduce costs and continue to improve prognostic, omics data are promising. With the decreasing price of RNA sequencing (RNA-seq), the accessibility of affordable technologies [e.g., MinION from Oxford Nanopore Technologies (Menegon et al., 2017)], the available PCa cohorts and the efficient computational approaches, transcriptomics is becoming a valuable resource to identify biomarkers (Nikitina et al., 2017). The rapid development of omics technology has led to the availability of many omics databases (Marx, 2013; Almeida et al., 2014; Stephens et al., 2015), including The Cancer Genome Atlas Program (TCGA) (Tomczak et al., 2015) and those of the International Cancer Genome Consortium (ICGC) (International Cancer Genome Consortium Hudson et al., 2010), thus opening an opportunity to apply and test machine learning algorithms (Li et al., 2016). These algorithms have been utilized as an aim to model the progression and treatment of cancerous conditions, and resulted in effective and accurate decision-making (Kourou et al., 2015). However, many of the datasets results from patients cohorts that were either rather small and/or had insufficient follow-up of clinical history which limit their use for clinical outcome prediction.

Hence, there is a challenge to set up predictive models that could anticipate the event of BCR, thus predicting the evolution of cancer, immediately after surgery. Consequently, we propose here a method to discover a transcriptomic signature that could be used to predict BCR events using a combination of datasets to increase the discovery potential. To this purpose, we applied specific preprocessing and cleaning steps on three RNA-seq datasets and established a machine learning protocol.

## Materials and Methods

### Research Pipeline

**FIGURE 1:**
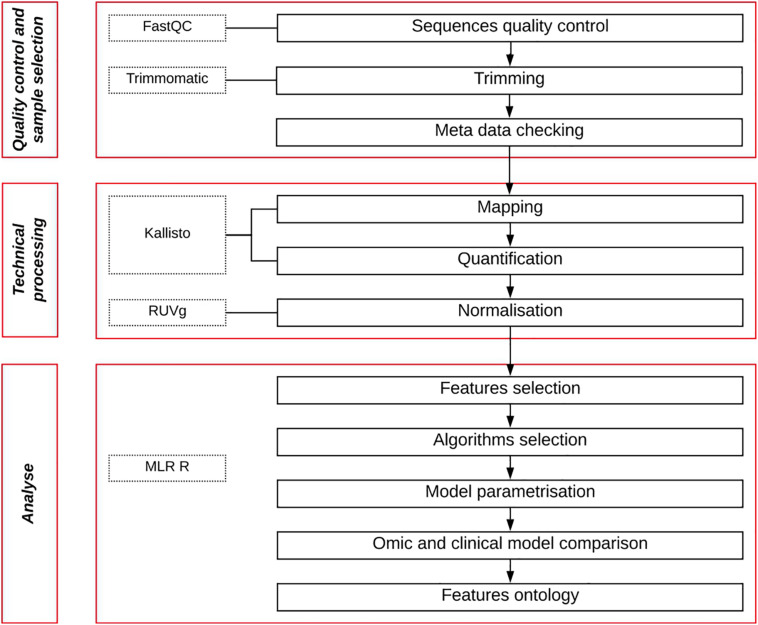
Pipeline workflow. Quality control of raw data sequencing files is measured, then trimmed to remove their adaptors. Patient metadata are then filtered to keep only BCR patients with long follow-up. Retained sequences are then mapped, quantified and normalized. Finally, a machine learning approach is used to analyze the data to obtain a gene expression predictive signature and a model.

After recovering the raw data from the different studies, we processed them in a pipeline composed of three main steps: Samples quality control and selection, sequencing data processing, machine learning analysis (Figure 1). All developed scripts are available in the github repository (See section “Data Availability Statement”).

### Datasets

We retrieved three different RNA-Seq datasets of radical prostatectomy specimens with the associated clinical features. The first dataset is from TCGA cohort in the Prostate Adenocarcinoma (PRAD) project. The second dataset (GSE54460) is from a cohort constituted by Long et al. (2014) and the third dataset was provided by Prof C. Collins from the Vancouver Prostate Cancer Center (VPCC) (Wyatt et al., 2014).

Quality of the BCR event data is dependent on patient clinical follow-up. A patient followed only a few weeks or months after surgery without showing BCR would be considered as a non-BCR case. These cases are a bias since the patient could have experienced a BCR event after the period of follow-up. Consequently, we discarded from our analysis the patients with no BCR whose follow-up was inferior to 60 months.

#### TCGA-PRAD Dataset

Data from 498 samples were initially recovered from the PRAD project on the TCGA data portal^1^. According to the *TCGA Research Network* (Cancer Genome Atlas Research Network, 2015) 131 samples must be discarded because of the presence of RNA degradation, as we did. We also ignored samples with less than 40% of tumor cells (column *percent_tumor_cells* in clinical file) and follow-up inferior to 60 months. We ended up with 52 samples after these filters.

#### GSE54460 Dataset

The data were downloaded from NCBI website (GEO accession GSE54460) where sequencing and clinical data from 106 patients were recovered. After selecting cases with a minimum of 60 months of follow-up, we ended-up with 96 patients of whom 54 had a BCR.

#### VPCC Dataset

We obtained the raw fastq files and clinical data from 85 patients, available at European Nucleotide Archive of the EMBL-EBI under accession PRJEB6530. Patients treated with hormonal therapy before radical prostatectomy were removed because this treatment strongly alters RNA expression. After selecting patients for minimal follow-up we ended up with 23 patients of whom five experienced a BCR.

The baseline characteristics of the resulting individual and combined cohorts after selection of eligible cases are summarized in Table 1.

**TABLE 1:** Baseline characteristics of the cohorts.

		TCGA	GSE54460	VPCC	Total
**Patients**					
		52	96	23	171
**Grade**					
*Low grade*					
5		0	1	3	4
6		2	9	12	23
7		14	72	4	90
	*Total*	16	82	19	117
**High grade**					
8		9	9	1	19
9		27	5	2	34
10		0	0	1	1
NA		0	0	0	0
	*Total*	36	14	4	54
	Total	52	96	23	171
**Stage**					
T1C		0	14	0	14
T2		0	7	0	7
T2A		1	21	3	25
T2B		2	10	0	12
T2C		9	26	17	52
T3		0	2	0	2
T3A		16	5	2	23
T3B		24	9	1	34
T4		0	1	0	1
NA		0	1	0	1
	Total	52	96	23	171
**BCR**					
NO		14	54	5	73
YES		38	42	18	98
	Total	52	96	23	171
**PSA at dx/preop**					
< = 10		31	64	21	116
10–20		16	17	1	34
> = 20		5	12	1	18
NA		0	3	0	3
	Total	52	96	23	171

### Quality Control, Alignment and Gene Expression

**FIGURE 2:**
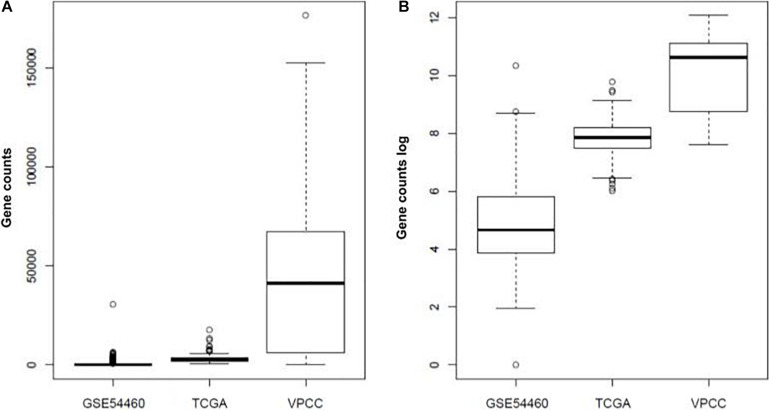
Summary of gene expression value in each dataset **(A)** or log of the expression value **(B)**.

The quality of the raw fastq files from the TCGA cohort was measured using *FastQC* (Andrews et al., 2010) (v0.11.5) and *Trimmomatic* (Bolger et al., 2014) (v0.32). A threshold quality per base of 30 (based on Phred 33) and a minimal length of 40 bases were applied. The transcriptomes were then mapped on GrCH38.p7 using *Kallisto* (Bray et al., 2016) (v0.43.0). The software Kallisto was used to estimate isoform counts, adjusted for the amount of bias in the experiment to ensure a coherent no-naive mapping. Default paired end parameters indicated in kallisto’s manual were used. The index needed to run Kallisto is provided on the official github repository^2^, but can be manually created. Consequently, we computed gene counts with *tximport* (Soneson et al., 2015) (Figure 2). The Ensembl gene identifiers were converted with *Biomart tools* (Kinsella et al., 2011; Smedley et al., 2015) from transcript ID to gene ID. For both GSE54460 and VPCC datasets, we processed the raw fastq files using the same method as for the TCGA dataset. However, in GSE54460 the ribosomal sequences were still present within the reads, so we separated these sequences from the mapped reads and removed them. After mapping procedure, 29820 Ensembl genes were found in TCGA-PRAD dataset, 28704 in GSE54460 dataset and 32334 in VPCC dataset. The difference of number of Ensembl genes detected is explained by the sequencing depth of the datasets. A total of 25504 Ensembl genes were common to all sets and were retained for the analysis.

### Normalization

The gene expression data were normalized with the *RUV method* (Gagnon-Bartsch and Speed, 2012; Risso et al., 2014) in each dataset separately following the default protocol indicated in the RUVseq package vignette. RUVg uses negative control genes [housekeeping genes (HKG)], assumed not to be differentially expressed. In order to normalize properly we selected in the literature a set of specific HKG candidates for PCa (de Kok et al., 2005; Ohl et al., 2005; Chua et al., 2011; Vajda et al., 2013): ACTB, PPIA, GAPDH, PGK1, GUSB, RRN18S, and RPL13A. The expression of these genes was tested by RT-qPCR in a series of 50 prostate tumors and the genes were shown to be stably expressed between tumor samples. We excluded from the final list the ribosomal genes RRN18S and RPL13A because ribosomal RNAs were removed from our RNA-seq datasets. PGK1 was also excluded according to recent results (Vajda et al., 2013). Finally, four genes were chosen: GUSB, PPIA, GAPDH, and ACTB.

### Machine Learning

**FIGURE 3:**
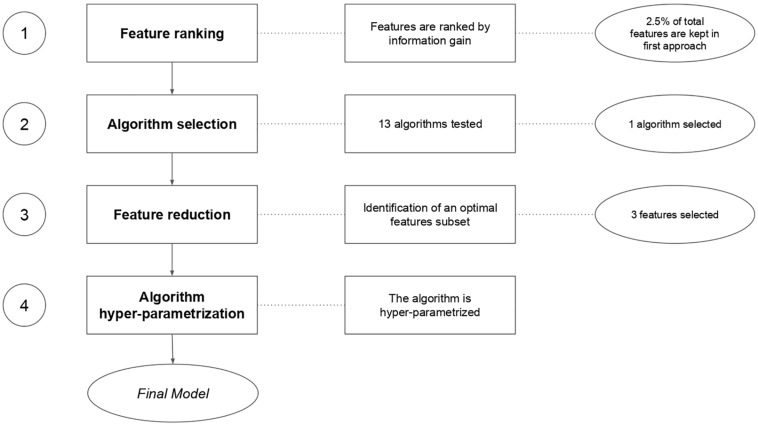
Machine learning feature selection and model evaluation workflow.

There are multiple approaches to treat biological data in a machine learning workflow (Al-Jarrah et al., 2015; Makridakis et al., 2018). Many machine learning libraries exist, in various programming languages, such as MLR in R (Lesmeister, 2015), Scikit-Learn (Garreta and Moncecchi, 2013) in python and WEKA (Hall et al., 2009) in Java. We chose the MLR (v2.8) package in R to set up our work. Our general workflow is described in Figure 3.

### Validation Strategy

We performed a resampling to assess the performance of the learning algorithm, avoid over-optimistic results and get a more robust measure of the performance of our model. The entire dataset was split into a random stratified (i.e., class balance preserved) training and testing sets, 1000 times, hence the classification algorithm is trained and tested on different sets. The measure of performance is an aggregated value (e.g., average) of the individual performance on the test set. Because we have no repeated measures and independent variables (i.e., the patients) we chose the subsampling method which is also the best in general in different benchmarks but is less effective computationally (Bischl et al., 2012). The resampling strategy was run 200 times with a split of 2/3 for training and 1/3 for test sets. In the resampling methods the split is usually 4/5 or 9/10. In our case we wanted to avoid over-optimistic results then we chose a smaller train set closer to a classical cross validation (CV) approach.

### Performance Metric

To evaluate the performance we used the balanced error rate (BER), the matthews correlation coefficient (MCC) and the mean misclassification error (MMCE). The BER is calculated as the average proportion of wrongly classified samples in each class and weights up small sample size classes (Table 2). The area under the curve (AUC) was also reported.

**TABLE 2:** Performance measures.

Performance metric	Formula
Sensitivity	TP/(TP + FN)
Specificity	TN/(TN + FP)
Accuracy	(TP + TN)/(TP + TN + FP + FN)^∗^100
MCC	$\sqrt{\left(\text{TP}+\text{TN}\right)/{\left(\text{TP}+\text{TN}+\text{FP}+\text{FN}\right)}^{*}\,100}$
BER	1–0.5 (Sensitivity + Specificity)
MMCE	Mean (response! = truth)

*The detailed formula of our metrics.*

### Feature Selection

Feature selection was performed to reduce dimensionality to improve prediction performances by removing uninformative features, which has been proven successful in other studies (Novakovic et al., 2011). There are different approaches to identify relevant features (Hira and Gillies, 2015; Singh and Sivabalakrishnan, 2015; Raza and Qamar, 2019). We chose information gain ranking, an entropy based method, that can handle both numerical (e.g., gene expression) and categorical data (e.g., clinical data). In MLR this method relies on the package FSelector which is an entropy based selection method (Lin, 1991; Coifman and Wickerhauser, 1992).

### Classifier Hyper-Parametrization

Algorithms typically require to change the settings of parameters to optimize their performance. The optimization method was the Irace method (López-Ibáñez et al., 2016) which is automated and implemented in an R package. We also work with a grid search algorithm for some specific parameters, which span the space in a number of chosen steps. These methods are also available within the MLR package to be used directly with the created tasks. The hyperparameters search depends on the algorithm iterated, defined in the MLR related man page.

## Results

### Model and Features Selection

**FIGURE 4:**
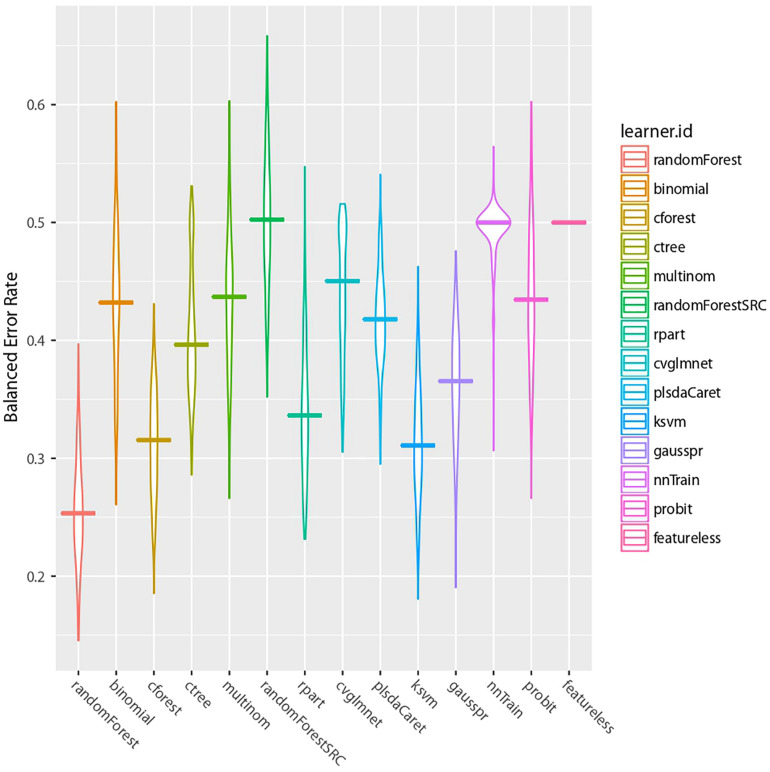
Machine learning algorithms comparison. The BER results of our 13 benchmarked algorithms are presented. The last model is a featureless control case.

Following our machine learning pipeline (Figure 3), we first reduced the dimension of the dataset and removed non-informative features to obtain 400 top ranked features to train and benchmark 13 models (Figure 4). We observed that the random forest (RF) algorithm (Ho, 1995) performed best on our data. The classical RF was chosen as the main model for our further analysis.

**FIGURE 5:**
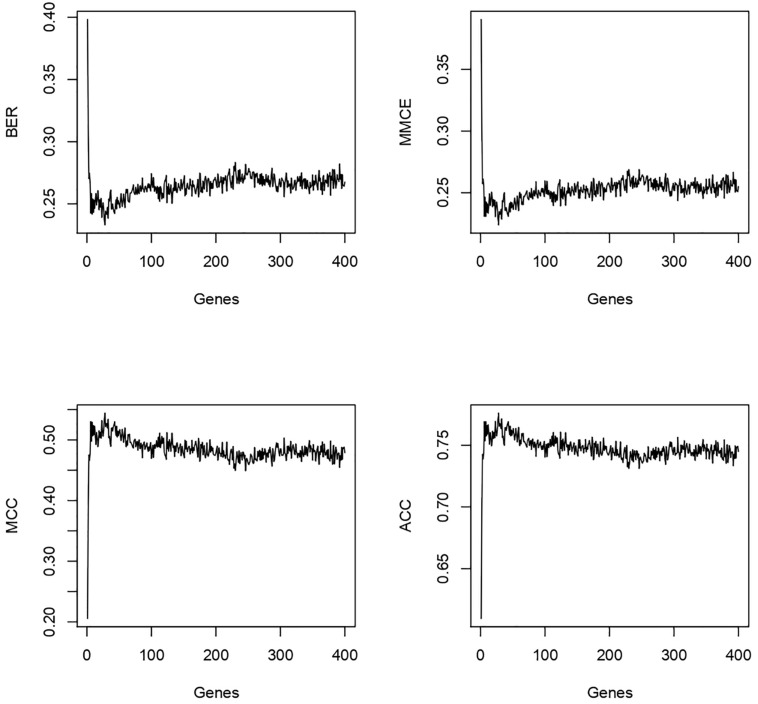
For the 400 genes tested the best genes/performance ratio is obtained with less than 20 genes in our model.

Since our goal was to identify a very short genomic signature we looked up the BER rate and other metrics while varying the number of selected features, from 1 to 400, used in the model. We observed that the BER and MMCE dropped rapidly with a few features selected (<3) then oscillated around 0.27 (Figure 5).

The MCC and the accuracy (ACC) went up rapidly and stabilized in the same way. After these observations, we focused the analysis on the first eight genes. The results are shown in Table 3. We observed a shift in BER value after adding the third most predictive gene to the signature. Afterward, BER begins to stabilize around 0.25–0.28 despite adding more informative genes. Consequently, we decided to keep the first three genes for the rest of the analysis. These genes are ENSG00000125534 (PPDPF), ENSG00000177606 (JUN), and ENSG00000188290 (HES4).

**TABLE 3:** Feature selection benchmark.

Nb of features	BER	MMCE	MCC	ACC	Gene name	ENSG
1	0.40	0.39	0.20	0.60	PPDPF	ENSG00000125534
2	0.32	0.30	0.38	0.69	HES4	ENSG00000188290
3	0.28	0.28	0.48	0.74	JUN	ENSG00000177606
4	0.28	0.26	0.47	0.73	GNB2	ENSG00000172354
5	0.28	0.26	0.48	0.74	PYROXD2	ENSG00000119943
6	0.25	0.23	0.53	0.77	MAP3K2	ENSG00000169967
7	0.27	0.25	0.50	0.75	RPL28	ENSG00000108107
8	0.25	0.23	0.53	0.77	DHCR24	ENSG00000116133

*Benchmark on specific number of features has been performed and results of the performance metrics are presented.*

### Hyper-Parameters Optimization and Final Model

**FIGURE 6:**
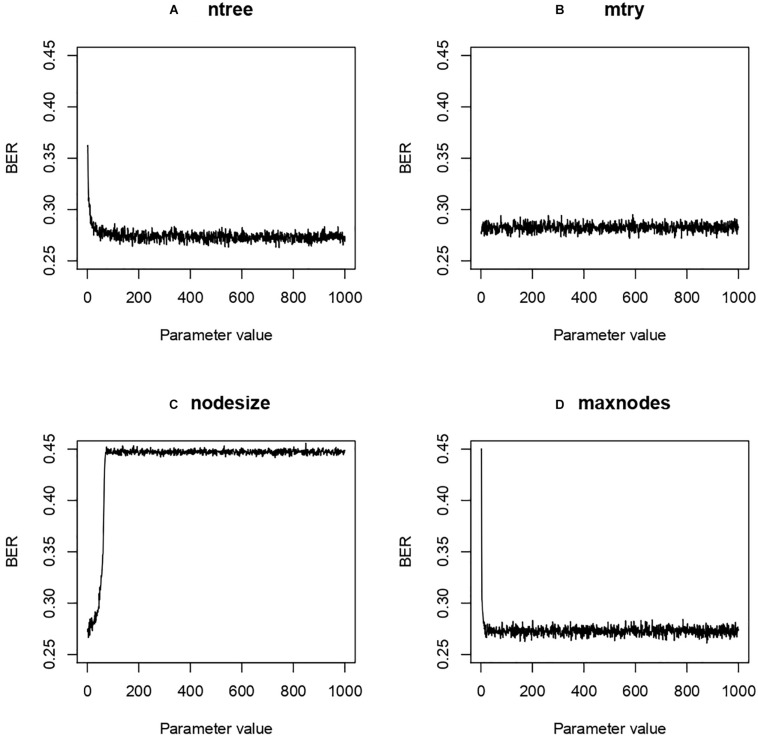
Balanced Error Rate (BER) evolution according to modulation of Random Forest (RF) parameters. Four different RF hyper-parameters were tested while keeping the others at default value in a grid search approach. The results were then used in an Irace search to find optimal parameters. **(A)** ntree, number of decision trees; **(B)** mtry, number of variables selected from a decision split for the next split; **(C)** maxnodes, maximal number of nodes; **(D)** nodesize, minimal number of samples allowed in a node.

Four hyper-parameters of the RF classifier were optimized: ntree, mtry, maxnode, and nodesize. Ntree refers to the number of decision trees in the model, mtry the number of variables selected from a decision split for the next split, maxnodes the maximal number of nodes in the forest and nodesize the minimal number of samples allowed in a node. Because we selected only three features, the parametrization step was not expected to drastically change the performance of our optimization task. First we used a grid search method to define the best setting for each parameter taken individually, letting the others at default. The grid search provided us 500 (ntree), 1 (mtry), 24 (maxnodes), and 5 (nodesize) (Figure 6).

From these hyper-parameters an Irace search was performed around the space of those values. The best value was obtained with ntree, mtry, maxnodes and nodesize at 187, 1, 881 and 1 resp. for a BER of 0.27. We observed relative stability despite the modification of the hyperparameters.

**FIGURE 7:**
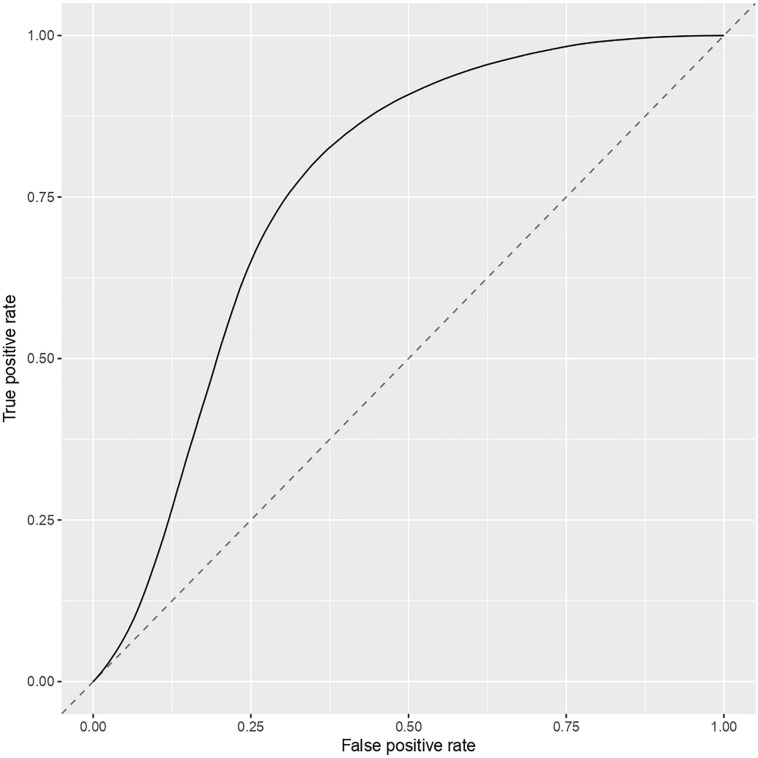
ROC curve for the three-gene model.

To ensure the stability of our three-gene model, a subsampling test was done 100000 times for the last part of our work. From this subsampling, the results obtained are ber = 0.274, mmce = 0.26, mcc = 0.468, fpr = 0.368, tpr = 0.82, acc = 0.739. Then we calculated the associated AUC (0.761) and plotted the ROC curve Figure 7.

**FIGURE 8:**
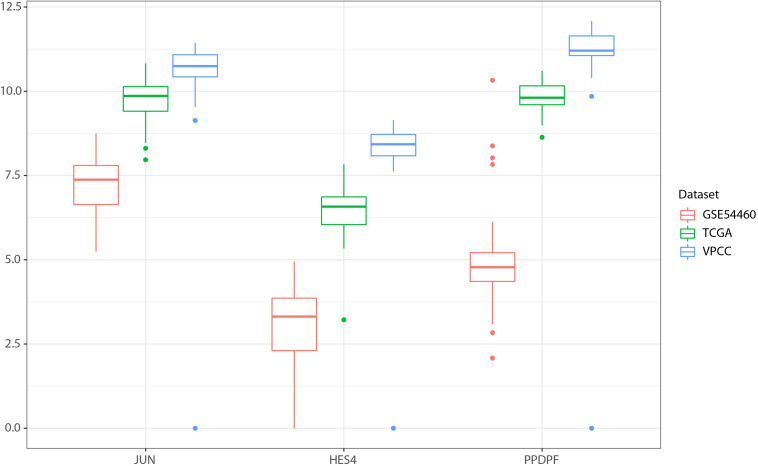
Log2 transformed distribution of normalized read counts for the three genes signature in each cohort.

The proposed three genes signature (see gene distribution for each cohort in Figure 8) model can be retrained using the training data provided in the github repository (see “Data Availability Statement” section), and new data must be processed following the indications in Materials and Methods before being submitted to the model.

### Comparison of Omics and Clinic Models

We compared the potential of omic data versus clinical data to assess the ACC of our omics model. A RF model for the clinical data (Grade, stage, and PSA) and a merged model combining clinic and omics data were set up following the same protocol used for the omics data. For the clinical model the best BER obtained was 0.311 and for the mixed model the best BER obtained was 0.276 (Table 4).

**TABLE 4:** Comparison of model performance using clinic or omics data or both.

Metric	Omics	Clinic	Omics + Clinic
BER	0.27	0.32	0.28
MMCE	0.257	0.306	0.265
MCC	0.474	0.373	0.457
ACC	0.742	0.692	0.734
**Parameters**			
ntree	187	1402	667
mtry	1	3	1
maxnodes	881	30	25
nodesize	1	4	6

*The omics model is based on three genes and the clinic model is a model based on the grade, stage and PSA. The omics + clinic model integrates all the selected features together.*

### Single Cohort Performance

**FIGURE 9:**
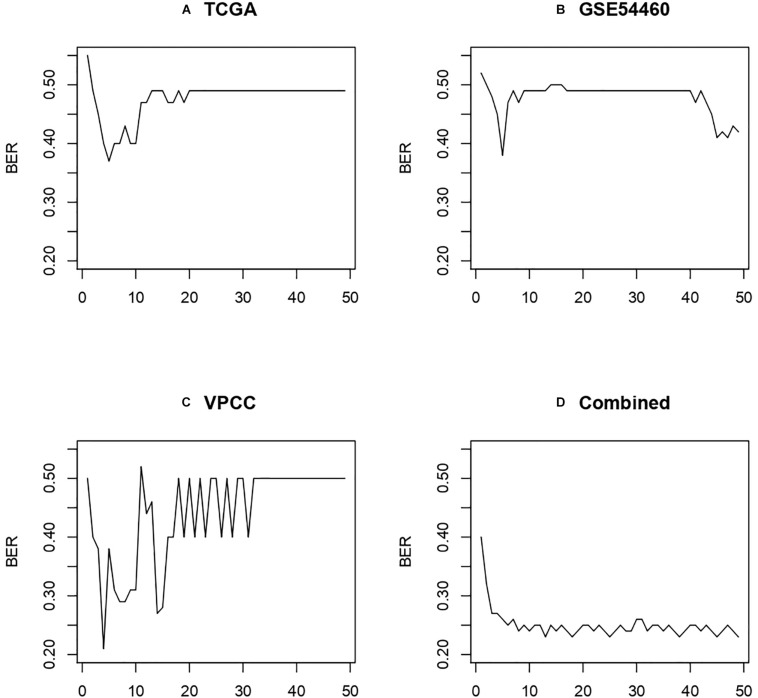
Performance obtained using leave one out group validation. **(A)** Model trained on GSE54460 and VPCC then tested on TCGA. **(B)** Model trained on TCGA and VPCC then tested on GSE54460. **(C)** Model trained on GSE54460 and TCGA then tested on VPCC. **(D)** Combined dataset evaluated by subsampling method described in “Validation Strategy.”

To further assess the performance of the three-gene model obtained with the combined dataset, we also performed the analysis with the individual cohorts. We used the RF algorithm iterated on the 50 best features from Information Gain on the three datasets evaluated by leave one out group validation (i.e., two datasets for training, one for testing), and the combined dataset evaluated by resampling (see section “Validation Strategy”). The results are displayed in Figure 9 and show that the combined dataset offers better and more stable performances.

## Discussion

Machine Learning is one of the fastest growing fields in bioinformatics (Inza et al., 2010) and its application to healthcare is a challenge. In the past decade, various mathematical methods using combination of omics biomarkers (Halabi et al., 2003; Gaudreau et al., 2016), including non-coding RNAs, PCA3, TMPRSS2:ERG (Nilsson et al., 2009) were developed to improve PCa diagnosis (Wang et al., 2017; Guo et al., 2018), define the grade (Arvaniti et al., 2018), define the risk (Paulo et al., 2018) and predict survival time (Zupan et al., 2000). Machine learning approaches to predict BCR or other characteristics demonstrated good performances in various situations. Lalonde et al. (2014, 2017) built a 100 loci-DNA (CNV) signature for low to high risk cohort with 563 patients and a 60-month follow-up for BCR. The obtained AUC was 0.74, which is similar to our performance but with another technology (CNV assay) and for much fewer biomarkers. Moreover, a model containing so many features can be suspected of overfitting. Regnier-Coudert et al. (2012) built a model on Partin table from a large cohort of 1700 patients to improve cancer grading and staging, and obtained an AUC of 0.68. Mangiola et al. (2018) focused on gene expression but chose to predict dichotomous cohorts with low versus high risk patients. With a cohort of 80 patients and an average follow-up of 27–29 months they achieved an AUC of 0.72. Finally, Abou-Ouf et al. (2018) used a large cohort of 545 patients to define a ten-gene signature from microarray exon chips to predict BCR, but couldn’t exceed an AUC of 0.65. Thus, there was a large room for improvement in terms of predictive performance, and a lack of focus on small gene signature, much easier to reproduce, to predict BCR with recent technology (RNA-Seq).

In this study, we took advantage of the power of machine learning to identify a biomarker signature composed of three genes. We showed that such short signature from omics data performs better to predict BCR than clinico-pathological features or a combination of these data (i.e., clinico-pathological + omics data). We have explored many machine learning algorithms, since each has its advantages and drawbacks in terms of computational time, hyper-parameters and range of application (class, type and dimension) and also because their performance depends on the type of data and their composition (Heung et al., 2016). Using this approach, we ended with a Random Forest model with a 27% BER with a three genes signature.

The identified signature contains three genes: JUN, HES4, and PPDPF. Gene JUN is well known for being a transcription factor acting as an oncogene (Maki et al., 1987; Vogt and Bos, 1990; Wasylyk et al., 1990; Mariani et al., 2007). Proteins of the JUN family combined with the Fos protein to form the heterodimeric AP-1 transcription factor. This complex can enter into the nucleus and bind specific DNA sequences to module targeted genes. AP-1 activity is induced by stimuli such as growth factors and cytokines that bind to specific cell surface receptors (Yang et al., 1999). Recently a miRNA targeting JUN has been identified as tumor suppressor (Liu et al., 2015).

*Hes Family BHLH Transcription Factor 4* (HES4) is a gene related to the PI3K-Akt signaling pathway. This gene is a transcription factor binding DNA. It is related to the NOTCH3 receptor and is a biomarker of PCa aggressiveness (Carvalho et al., 2012) and is also related to colorectal cancer in the same pathway (Sikandar et al., 2010). It was demonstrated as a high grade biomarker of osteosarcoma (McManus et al., 2017).

Finally, PPDPF is known to be expressed during pancreas development [Pancreatic Progenitor Cell Differentiation And Proliferation Factor (Breunig et al., 2017)] and differentially expressed in several types of cancer (Voena et al., 2013; Xue et al., 2015). But it was not previously associated with PCa.

We have attempted to understand the biological links between these three genes and the eventual relation with the BCR. This is not straightforward considering that Random Forest models tend to reflect a nonlinear approximation of statistical relationships, hence providing little insight of how elements of the signature are related. Thus, we have performed a protein-protein interaction networks functional enrichment analysis using String-DB (Szklarczyk et al., 2019) on the three identified genes, but no evident relations could be found, even after addition of intermediate protein nodes. We have also performed a gene list enrichment analysis and candidate gene prioritization based on functional annotations using ToppGene Suite (Chen et al., 2009) using the three identified genes. The only biologically relevant (i.e. cancer hormono-dependant as the PCa) and significant (*q*-value 2.1E-2 after FDR Benjamini-Yekutieli procedure correction) hit is that the three genes exist in the Human Breast Nam08 30 genes UpregulatedGeneList signature (Nam et al., 2008), provided by GeneSigDB (Culhane et al., 2012), but no evident and/or significant biological functions by ontology seem to link these three genes together. We have eventually expanded the list of three genes to 320 genes by retrieving correlated genes (>90% Pearson correlation) and observed that many genes were involved in mitochondrial functions, including mitochondrial translation, mitochondrial gene expression, mitochondrial translational termination and mitochondrial translational elongation, all having a *q*-value <5.9E-5 after FDR Benjamini-Yekutieli procedure correction. This observation is supported by other studies who have found a clear relation between mitochondrial genomic alterations and BCR (Ellinger et al., 2008; Kalsbeek et al., 2016; Xu et al., 2020).

This is not the first time that predictive three-genes signatures have been identified in various diseases (Sun et al., 2015; Thakkar et al., 2015; De Palma et al., 2016; Ibrahim et al., 2016; Wang et al., 2016; Li et al., 2017; Chen et al., 2018; Yang et al., 2018; Bao et al., 2019; Ding et al., 2019; Saidak et al., 2019; Xiao et al., 2020), hence showing that extensive research is ongoing to identify multigenic signatures containing a reasonable number of potential targets. The identified genes could be eventually verified in other cohorts or by experimental validations. One key point should be to add gradually smaller datasets to control the signature stability with various experiments and technologies. Integrate too large cohorts in this approach will imbalance model parameters in favor of that cohort, then all the advantages of using several small dataset will be lost. This approach has the advantage of offering a small research team the opportunity to integrate their own work in a larger view. After integrating more dataset, a set up in a specific technology such as TaqMan probe to evaluate gene expression could be proposed as diagnosis and maybe to develop drugs (Laetsch et al., 2018; Havel et al., 2019).

## Conclusion

By using an appropriate data transformation strategy and machine learning pipeline, we have identified a three-gene signature. With the decreasing price of RNA sequencing and its growing accuracy there are opportunities for less invasive and faster exams if the right biological variables are chosen. Other investigations on other omics data using the same machine learning approach could be undertaken, such as using miRNAs (Kristensen et al., 2016; Matin et al., 2018). We also showed that it is possible to concatenate several cohorts to get stable and performing models from heterogeneous RNA-Seq PCa datasets, hence showing a robustness against batch effect. This study demonstrates the potential of taking advantage of many independent datasets produced on the same disease. Machine learning algorithms can handle the batch effect if there is the right preprocessing pipeline applied on the data.

## Data Availability Statement

Publicly available datasets were analyzed in this study. This data can be found here: TCGA at GDC data portal; GEO accession GSE54460; The European Nucleotide Archive (ENA), accession number PRJEB6530 from Wyatt et al. (2014). Moreover, the scripts developed for this study and the processed read counts are available at github.com/ArnaudDroitLab/prostate_BCR_ prediction.

## Ethics Statement

This study was approved by the Research Ethics Committee of the CHU de Québec-Université Laval (Project 2018-3670). Written informed consent for participation was not required for this study in accordance with the national legislation and the institutional requirements.

## Author Contributions

BV conducted literature searches, gathered the data, wrote the code to perform the research, and wrote the manuscript. He also created figures and tables, and wrote, formatted the manuscript for submission. ML participated to design the approach. ML, M-LM-M, and AB helped to improve the manuscript. YF and AD supervised and reviewed the design of the study. CC provided the VPCC data. All authors contributed to reviewing the manuscript.

## Conflict of Interest

The authors declare that the research was conducted in the absence of any commercial or financial relationships that could be construed as a potential conflict of interest.

## Footnotes

1 https://portal.gdc.cancer.gov/

2 https://github.com/pachterlab/kallisto-transcriptome-indices/releases

## Abbreviations

ACC: accuracy
BER: balanced error rate
BCR: biochemical recurrence
AUC: area under the curve
MCC: matthews correlation coefficient
MMCE: mean misclassification error rate
PCa: prostate cancer
PSA: prostate specific antigen
TNM: tumor node metastasis.
